# *Lactiplantibacillus plantarum* OLL2712 Protects From the Intestinal Dysfunction in D‐Galactose Induced Senescent Cells

**DOI:** 10.1002/fsn3.71019

**Published:** 2025-09-28

**Authors:** Yumiko Watanabe‐Yasuoka, Reiko Watanabe, Ayako Gotou, Haruyo Nakajima‐Adachi, Satoshi Hachimura, Toshihiro Sashihara

**Affiliations:** ^1^ Wellness Science Labs Meiji Holdings Co., Ltd. Tokyo Japan; ^2^ Health Science Research Unit, Division of Research and Development Meiji Co., Ltd. Tokyo Japan; ^3^ Research Center for Food Safety, Graduate School of Agricultural and Life Sciences The University of Tokyo Tokyo Japan

**Keywords:** antioxidant, cellular senescence, D‐galactose, intestinal barrier function, *Lactiplantibacillus*

## Abstract

Cellular senescence refers to a state in which cells stop dividing and can no longer proliferate because of various factors, such as DNA damage and oxidative stress. It is implicated as a key driver of aging and age‐related diseases. *Lactiplantibacillus plantarum* OLL2712 (OLL2712) is a lactic acid bacterium that alleviates chronic inflammation and improves the intestinal barrier and cognitive functions. These effects are important for preventing the changes that occur in the body with aging. In this study, we hypothesized that OLL2712 protects against intestinal barrier dysfunction associated with cellular senescence. To reveal the antiaging effect of OLL2712, we used D‐galactose (D‐gal) to induce cellular senescence in Caco‐2 cells and investigated its effects on various phenomena occurring in senescent intestinal cells. The results showed that OLL2712 suppressed D‐gal‐induced cellular senescence and intestinal barrier dysfunction. Furthermore, microarray analysis showed that OLL2712 upregulated signaling involved in antioxidant effects, metabolism, and cell division, which were downregulated by cellular senescence. Overall, OLL2712 maintained intracellular homeostasis and restored intestinal barrier dysfunction associated with cellular senescence. We discovered the possibility that paraprobiotics contribute to the antiaging effects.

## Graphical abstract

*Lactiplantibacillus plantarum* OLL2712 (OLL2712) on intestinal dysfunction related to cellular senescence was investigated. Using D‐galactose to induce senescence in Caco‐2 cells, OLL2712 suppressed cellular senescence and restored barrier integrity, while microarray analysis revealed it upregulated signaling pathways related to antioxidants and metabolism. Our findings suggest that OLL2712 is likely to contribute to anti‐aging effects by maintaining intracellular homeostasis in senescent intestinal cells.

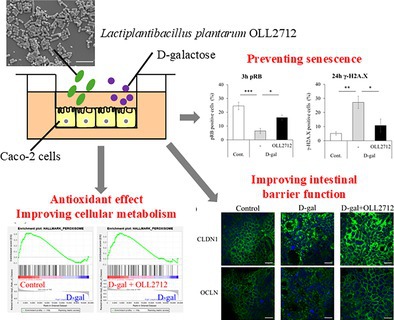

## 1 Introduction

Aging refers to a decline in physiological functions that occur with age. According to the United Nations World Social Situation Report 2023, the world population aged over 65 years is expected to more than double from 761 million in 2021 to 1.6 billion in 2050 (United Nations 2023). Staying healthy is important from a social‐welfare perspective. Many diseases, including Alzheimer's disease, type 2 diabetes, and atherosclerosis, occur with age (Childs et al. 2015; Liu 2022). Age‐related diseases develop in various organs throughout the body, and characteristic changes also appear in the intestinal tract.

It has been reported that phenotypic changes in intestinal epithelial cells associated with aging in rats include a decrease in the thickness and height of microvilli and a decrease in the expression of tight junction (TJ)‐related genes, which impair the intestinal barrier function (Ren et al. 2014). Studies using mice and human intestinal organoids have revealed that the function of intestinal stem cells, which differentiate into various intestinal epithelial cells, decreases with age (Nalapareddy et al. 2017). In humans, studies using an Ussing chamber have shown that biopsy samples of the small intestine from elderly individuals show increased permeability, including decreased trans epithelial electrical resistance (TEER) values and increased expression of the ion‐permeable protein, Claudin‐2 (CLDN2) (Man et al. 2015). The intestinal barrier insulates the body from the outside environment and helps to maintain intestinal homeostasis. We live symbiotically with commensal microorganisms in the intestine. When the intestinal barrier is disrupted, foreign substances derived from food and commensal or harmful microorganisms present in the food enter the body and cause inflammation throughout the body (Fukui 2016). Some lactic acid bacteria (LABs), commensal bacteria that are often used in fermented foods, improve the intestinal barrier (Kobayashi et al. 2023; Yu et al. 2012). This effect was also found in our research on our LAB strain, *Lactiplantibacillus plantarum* OLL2712 (OLL2712), in vivo and in vitro (Wang et al. 2023; Watanabe‐Yasuoka et al. 2023). This strain has also been shown to improve metabolic disorders by attenuating chronic inflammation through the induction of interleukin‐10 production in dendritic cells and macrophages (Takano et al. 2020; Toshimitsu et al. 2021, 2024, 2016). In addition, it has been reported that neuroinflammation in the brain and deterioration of insulin resistance are involved in the onset of Alzheimer's disease (Burillo et al. 2021). OLL2712 also alters gut microbiota involved in inflammation and improves cognitive function in the elderly (Sakurai et al. 2022). Therefore, OLL2712 is expected to be a LAB that contributes to the improvement of aging‐related intestinal barrier dysfunction.

Cellular senescence refers to a state in which cells stop dividing and can no longer proliferate due to various factors such as DNA damage and oxidative stress. It is directly implicated as a key driver of aging and age‐related diseases (Zhang et al. 2022). Experimentally, there are several methods that have been developed to induce cellular senescence. For example, WI‐38, a normal human fibroblast, first discovered the concept of cellular senescence and then underwent cellular senescence upon repeated passaging (Hayflick 1965). In the established cell lines, cellular senescence was artificially induced. D‐galactose (D‐gal) induces cellular senescence both in vivo and in vitro (Liu et al. 2017; Ru et al. 2022). It is normally metabolized to glucose; however, when added in excess, it cannot be decomposed and can be converted to hydrogen peroxide and aldose, inducing oxidative stress. It then reacts with amines of amino acids in proteins and peptides to produce advanced glycation end products and with free radicals and reactive oxygen species to cause cellular senescence (Anand et al. 2012). In this study, we investigated whether OLL2712 suppressed several age‐related phenomena in intestinal epithelial cells using Caco‐2 cells, considering animal welfare.

## 2 Materials and Methods

### 2.1 Preparation of Bacterial Cells

*Lactiplantibacillus plantarum* OLL2712, which was isolated in our laboratory as described previously (Toshimitsu et al. 2016) and deposited in the International Patent Organism Depositary (Chiba, Japan) under the accession No. FERM BP‐11262, was used in this study. *L. plantarum* JCM 1149^T^ was purchased from the Riken BRC (Ibaraki, Japan). They were cultured anaerobically with AnaeroPouch‐Anaero (Mitsubishi Gas Chemical, Tokyo, Japan) in de Man, Rogosa, Sharpe broth (Becton Dickinson, Franklin Lakes, NJ, USA) at 37°C for 18 h. Bacterial cells were harvested, washed twice with phosphate‐buffered saline (PBS; pH 7.2), and washed once with distilled water. The cells were then heat‐treated at 75°C for 60 min and then freeze‐dried (Toshimitsu et al. 2016) for long‐term storage. Lyophilized cells were resuspended in distilled water at a concentration of 10 mg/mL and used for in vitro assays.

### 2.2 Cell Culture and Treatment

Caco‐2 cells, purchased from the European Collection of Authenticated Cell Cultures (Salisbury, UK), are widely used as small intestinal epithelial models. They were cultured in culture medium, high‐glucose Dulbecco's Modified Eagle Medium (Sigma‐Aldrich, St. Louis, MO, USA) supplemented with 10% fetal bovine serum (Biowest, Nuaille, France), 100 U/mL penicillin, 100 μg/mL streptomycin (Gibco, Waltham, MA, USA), and 1% minimum essential medium nonessential amino acid (MEM‐NEAA; Sigma‐Aldrich), in a 10‐cm dish and were maintained at 37°C in a humidified atmosphere containing 5% CO_2_. They were split at 80% confluence every 3 or 4 days. Using permeable supports (Transwell, 12‐ or 6.5‐mm diameter, 0.4‐mm pore size; Corning, Corning, NY, USA), the Caco‐2 cells were plated at a density of 9 × 10^4^ cells/cm^2^ and cultured for 3 weeks by changing the medium every 2 or 3 days. TEER was measured using the Millicell ERS‐2 system (Millipore, Burlington, MA, USA).

D‐(+)‐galactose (D‐gal; Fujifilm Wako Pure Chemical, Tokyo, Japan) was used to induce cellular senescence based on previous reports (Liu et al. 2017; Ru et al. 2022). It was dissolved in culture medium to a concentration of 30 mg/mL and filtered. When the cells were treated with both OLL2712 and D‐gal simultaneously, OLL2712 was added to the culture medium at a final concentration of 100 μg/mL determined by a previous report (Watanabe‐Yasuoka et al. 2023), which used OLL2712 on Caco‐2 monolayers, with the medium containing 30 mg/mL of D‐gal. Caco‐2 monolayers were treated with this medium for 1–3 or 24 h.

### 2.3 RNA Isolation and Quantitative Polymerase Chain Reaction (qPCR)

Total RNA from Caco‐2 cells was extracted using a Maxwell RSC48 automatic nucleic acid extractor (Promega, Madison, WI, USA) and a Maxwell RSC Simply RNA Cells Kit (Promega), following the manufacturer's instructions. RNA was quantified and assessed for purity using a NanoDrop (Thermo Fisher Scientific, Waltham, MA, USA). Complementary DNA was synthesized using the PrimeScript RT Master Mix (Takara Bio, Shiga, Japan), and PCR was performed using a GeneAmp PCR system 9700 (Applied Biosystems, Waltham, MA, USA). qPCR was performed using the KOD SYBER qPCR Mix (Toyobo, Osaka, Japan) and QuantStudio 3 Real‐time PCR system (Applied Biosystems) according to the manufacturer's protocol. The primer sets used are listed in Table 1. Amplification conditions were as follows: pre‐denaturation at 98°C for 2 min, denaturation at 98°C for 10 s, annealing at 60°C for 10 s, extension at 68°C for 30 s, a total of 40 cycles. mRNA expression was normalized to that of the housekeeping gene, glyceraldehyde 3‐phosphate dehydrogenase (*GAPDH*).

**TABLE 1:** The sequence of primers for qPCR.

Gene	Direction	Sequence (5′→3′)
*p53*	Forward	ACAAGGTTGATGTGACCTGGA
	Reverse	TGTAGACTCGTGAATTTCGCC
*p21* (Okumura et al. 2021)	Forward	AGCGATGGAACTTCGACTTTG
	Reverse	CGAAGTCACCCTCCAGTGGT
*NQO1*	Forward	GAAGAGCACTGATCGTACTGGC
	Reverse	GGATACTGAAAGTTCGCAGGG
*GPX2*	Forward	GGTAGATTTCAATACGTTCCGGG
	Reverse	TGACAGTTCTCCTGATGTCCAAA
*SOD2*	Forward	GCTCCGGTTTTGGGGTATCTG
	Reverse	GCGTTGATGTGAGGTTCCAG
*GAPDH*	Takara, HA067812	

*Note:* Not referenced PCR primer sequences were obtained from PrimerBank (https://pga.mgh.harvard.edu/primerbank/) (Spandidos et al. 2010).

### 2.4 Immunofluorescent Staining

After Caco‐2 monolayers were stimulated with D‐gal or D‐gal + OLL2712, they were fixed in 4% paraformaldehyde for 10 min at room temperature. The cells were washed with PBS and permeabilized with PBS containing 0.2% Triton X‐100 (PBS‐T) for 15 min at room temperature. Cells were blocked with 2% normal goat serum for 20 min and then incubated with primary antibodies anti‐Phospho‐Rb (Ser807/811) (8516T, 1:1000; Cell Signaling Technology, Danvers, MA, USA), anti‐Phospho‐Histone H2A.X (Ser139) (9718T, 1:500; Cell Signaling Technology), anti‐Claudin‐1 (NBP2‐61630, 1:200; Novus Biologicals, Centennial, CO, USA), anti‐Occludin (GTX114949, 1:200; GENETEX, Irvine, CA, USA), and anti‐ZO‐1 (GTX108627, 1:100; GENETEX) for 2 h at room temperature. After washing five times with PBS‐T, the cells were incubated with the appropriate fluorescein‐conjugated secondary antibodies (ab150077, ab150117; Abcam, Cambridge, UK) for 1 h at room temperature. Actin staining was also performed to identify senescent cell markers. A phalloidin‐iFluor 647 conjugate (20555; Cayman Chemical, Ann Arbor, MI, USA) was incubated with the secondary antibody. After washing five times with PBS‐T, the cells were mounted, and the nuclei were stained with 4′,6‐diamidino‐2‐phenylindole (DAPI; Thermo Fisher Scientific). Images were captured using a fluorescence microscope (Keyence, BZ‐X810, Osaka, Japan) or a confocal laser microscope (LSM880 model; Zeiss, Oberkochen, Germany). The percentage of senescence marker‐positive cells per field was counted automatically using a hybrid cell count application in the BZ‐X Analyzer software (Keyence).

### 2.5 Microarray and Gene Set Enrichment Analysis

The total RNA concentration was determined using an Agilent Bioanalyzer 2100 and RNA 6000 Nano LabChip Kit (Agilent Technologies, Santa Clara, CA, USA). Equivalent amounts of RNA from the same group were pooled at a final concentration of 100 ng. A GeneChip WT PLUS Reagent Kit (Thermo Fisher Scientific) was used to prepare the microarray samples according to the manufacturer's protocol. Samples were hybridized to Clariom S arrays for humans (Thermo Fisher Scientific) and scanned using the Affymetrix GeneChip Command Console installed on the GeneChip Scanner 3000‐7G. Gene expression was analyzed using Transcriptome Analysis Console software (Thermo Fisher Scientific).

Pathway analysis was performed using gene set enrichment analysis (GSEA; Broad Institute, Cambridge, MA, USA) (Mootha et al. 2003; Subramanian et al. 2005) based on Hallmark, Reactome, Kyoto Encyclopedia of Genes and Genomes (KEGG), and Gene Ontology Cellular Components (GOCC). Gene sets with a false discovery rate (FDR) *q*‐value < 0.25 were recognized as significantly enriched. *p* values < 0.05 were used to select the enriched gene sets when FDR *q*‐value > 0.25.

### 2.6 Statistical Analysis

Data are presented as mean value ± standard error. Statistically significant differences among groups were analyzed using Dunnett's multigroup comparison test in cases of equal variance. Otherwise, they were analyzed using a Steel's test. Statistical significance was set at *p* < 0.05. Statistical comparisons were performed using BellCurve for Excel ver 3.20 (Social Survey Research Information, Tokyo, Japan).

## 3 Results

### 3.1 D‐Gal Induces Senescence and OLL2712 Inhibits It in Caco‐2 Monolayers

**FIGURE 1:**
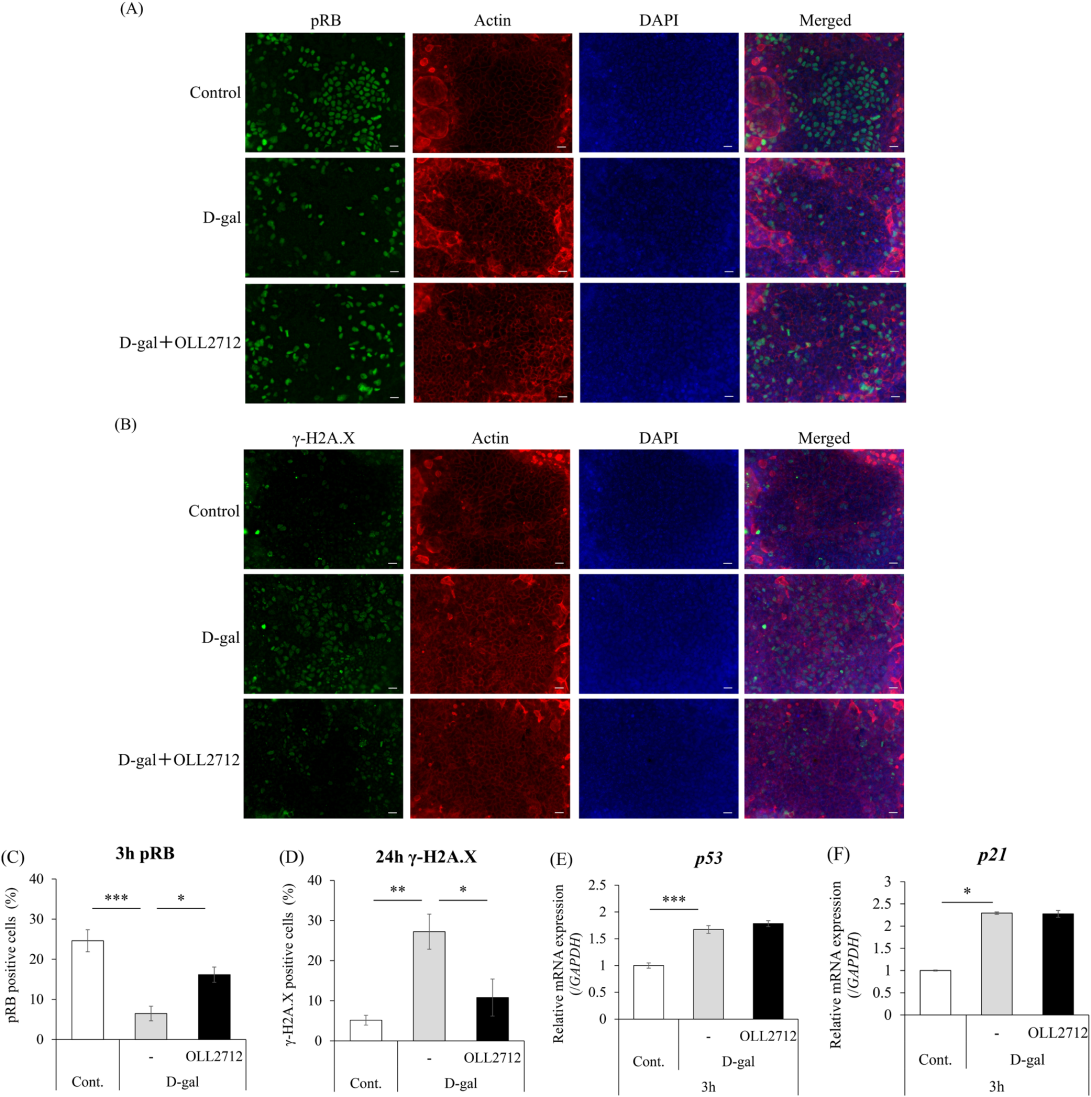
D‐gal induced cellular senescence and OLL2712 suppressed it in Caco‐2 monolayers. Caco‐2 monolayers were treated with D‐gal alone or in combination with OLL2712. (A, B) After 3 or 24 h of incubation, cellular senescence markers, phospho‐Retinoblastoma protein (pRB), and γ‐H2A.X, were visualized and analyzed via immunofluorescence microscopy (scale bar = 20 μm). (C, D) The number of pRB and γ‐H2A.X positive cells was counted (*n* = 5, 6). (E, F) After 3 h of incubation, the gene expression levels of the cellular senescence markers, *p53* and *p21*, were evaluated using quantitative polymerase chain reaction (qPCR). Data were normalized to glyceraldehyde 3‐phosphate dehydrogenase (*GAPDH*) expression and shown as relative expression (*n* = 4). Comparisons were performed using Dunnett's test or Steel's test. ***p < 0.001, **p < 0.01, *p < 0.05.

We first examined whether cellular senescence is induced in Caco‐2 monolayers by treating with D‐gal at a final concentration of 30 mg/mL for up to 24 h. Protein expression levels of phospho‐Retinoblastoma protein (pRB) and γ‐H2A.X that are used as markers of cellular senescence were analyzed by immunofluorescent staining (Lu et al. 2023; Min et al. 2022). RB is phosphorylated Ser807/811 during cell cycle progression, so the level of pRB is reduced during cellular senescence, whereas H2A.X is phosphorylated Ser139 and changed to γ‐H2A.X when DNA is damaged (Sherr 1996; Yuan et al. 2010). The results showed that the number of pRB positive cells was decreased at 3 h of D‐gal treatment compared with that in the control group, and the number of γ‐H2A.X positive cells was increased at 24 h of D‐gal treatment. However, when OLL2712 was simultaneously treated with D‐gal, these responses were suppressed (Figure 1A–D). It suggested that D‐gal induces cellular senescence in Caco‐2 cells, even in the static state as a monolayer, and OLL2712 suppresses cellular senescence, based on expression levels of pRB and γ‐H2A.X. However, when we examined the gene expression of *p53* and *p21*, which are other cellular senescence markers, their expression levels were increased by D‐gal but were not suppressed by OLL2712 (Figure 1E,F).

### 3.2 OLL2712 Improves Intestinal Barrier Dysfunction Related to Cellular Senescence

**FIGURE 2:**
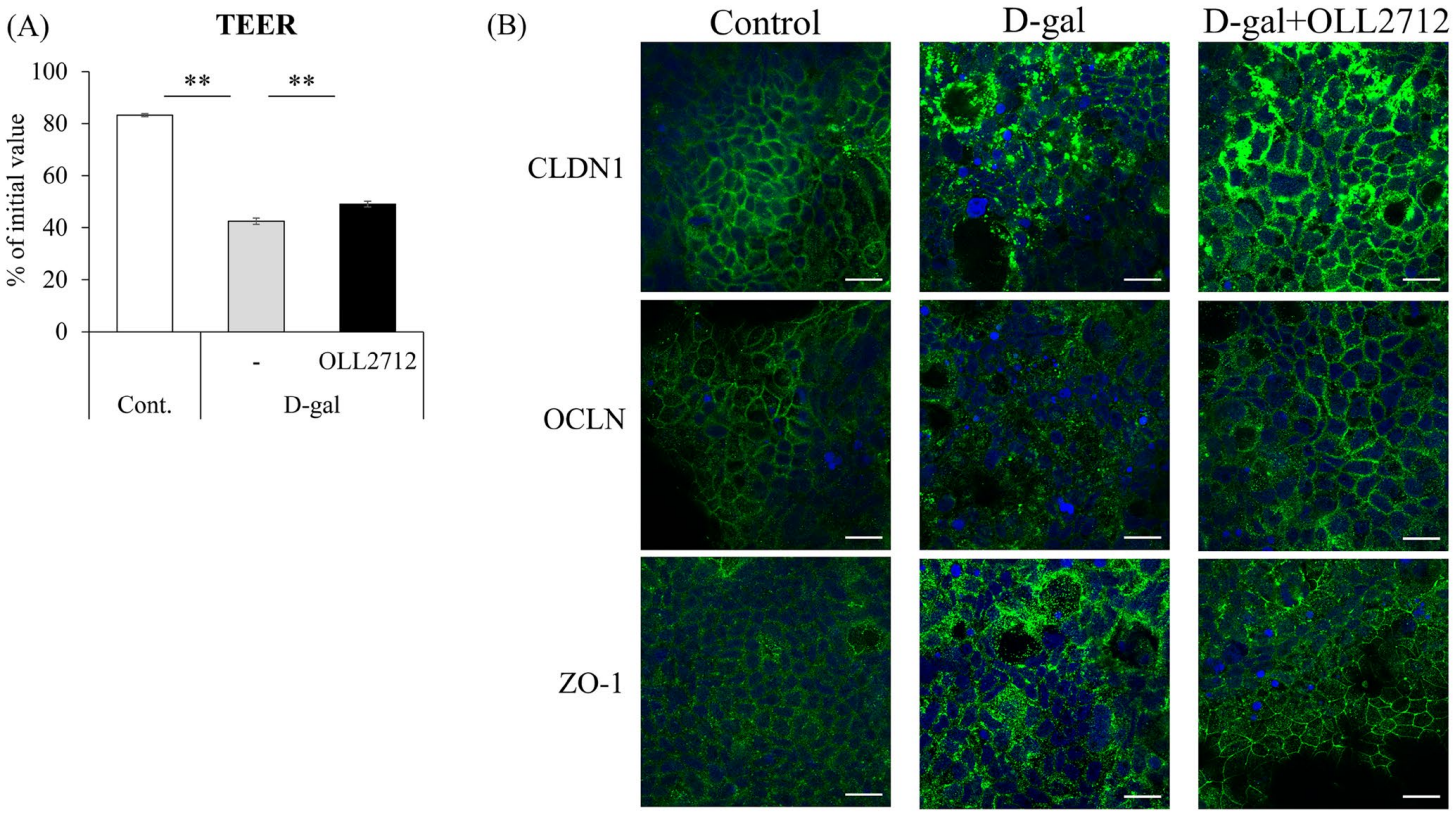
OLL2712 improved cellular senescence related to intestinal barrier dysfunction. Caco‐2 monolayers were treated with D‐gal alone or with D‐gal and OLL2712 simultaneously for 3 h. (A) Transepithelial electrical resistance (TEER) was measured (*n* = 4, 5). Comparisons were performed using Dunnett's test. **; *p* < 0.01. (B) Tight junction related proteins (claudin [CLDN]‐1, occludin [OCLN], zonula occludens‐1 [ZO‐1]) were visualized using a confocal laser scanning microscope (scale bar = 20 μm).

D‐gal treatment induces cellular senescence as well as reduces intestinal barrier function in vivo and in vitro (Ru et al. 2022; J. Wang et al. 2022). We evaluated the effect of treatment with D‐gal and OLL2712 for 3 h on the intestinal barrier function in Caco‐2 monolayers. The TEER values and protein levels of the TJ‐associated proteins CLDN1, occludin (OCLN), and zonula occludens‐1 (ZO‐1) were compared. TEER values were significantly decreased by D‐gal compared with those in the control group and significantly restored by OLL2712 (Figure 2A). Because another *L. plantarum* strain showed a comparable effect (Yu et al. 2023), we examined whether *L. plantarum* subsp. *plantarum* JCM 1149^T^, the type strain of *L. plantarum* , would have a similar impact. The result showed that it also suppressed the decrease in TEER value and restored D‐gal induced barrier dysfunction (Figure S1). Furthermore, immunofluorescence staining revealed that the structures of TJ‐associated proteins were disrupted by D‐gal and restored by OLL2712 (Figure 2B). It is suggested that OLL2712 restores the intestinal barrier dysfunction associated with cellular senescence, and the effect may not be limited to OLL2712.

### 3.3 OLL2712 Has Antioxidant Effects

**FIGURE 3:**
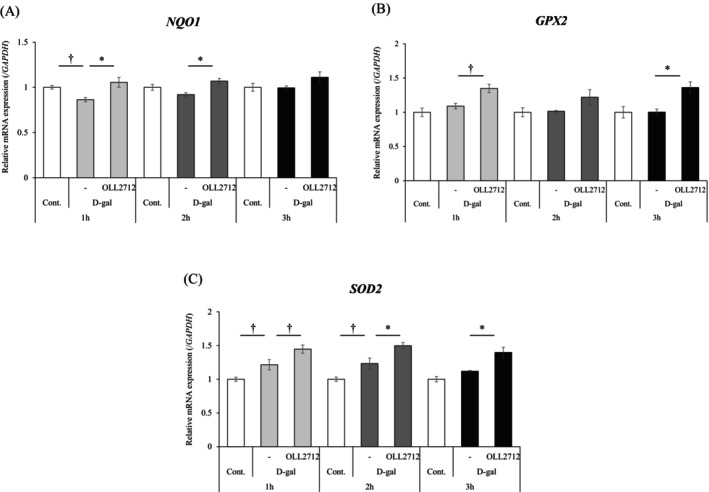
OLL2712 has an antioxidant effect in senescent cells. Caco‐2 monolayers were treated with D‐gal alone or with D‐gal and OLL2712 simultaneously for 1–3 h. Gene expression levels of anti‐oxidative stress markers (A) (NAD(P)H quinone dehydrogenase 1 (*NQO1*), (B) glutathione peroxidase 2 (*GPX2*), and (C) superoxide dismutase (*SOD2*)) were evaluated by qPCR. Data were normalized to glyceraldehyde 3‐phosphate dehydrogenase (*GAPDH*) expression and are shown as relative expression (*n* = 3, 4). Comparisons were performed using Dunnett's test. *; *p* < 0.05, †; *p* < 0.1.

The suppression of oxidative stress is important for antiaging effects (Liguori et al. 2018). The mechanism by which D‐gal induces cellular senescence is initiated by oxidative stress; therefore, we hypothesized that OLL2712 suppresses cellular senescence through its antioxidant effects. Caco‐2 monolayers were treated with D‐gal and OLL2712 for 1–3 h. We measured the gene expression levels of several antioxidant markers using qPCR. NAD (P) H quinone dehydrogenase 1 (*NQO1*) showed a tendency to decrease after 1 h of treatment with D‐gal but was significantly restored by OLL2712 (Figure 3A). On the other hand, glutathione peroxidase 2 (*GPX2*) and superoxide dismutase 2 (*SOD2*) were not decreased in the D‐gal group but showed the highest levels in the OLL2712 group (Figure 3B,C). It was suggested that OLL2712 possesses antioxidant properties.

### 3.4 OLL2712 Ameliorates Metabolic Change Related to Cellular Senescence

We demonstrated that OLL2712 suppressed cellular senescence‐related phenomena, such as the expression of cellular senescence marker protein levels, oxidative stress, and intestinal barrier dysfunction. We performed microarray transcriptome analysis to comprehensively analyze the cellular responses during the induction of cellular senescence and treatment with OLL2712. Caco‐2 monolayers were treated with D‐gal and OLL2712 for 3 h. Total RNA was extracted from cells and analyzed.

**FIGURE 4:**
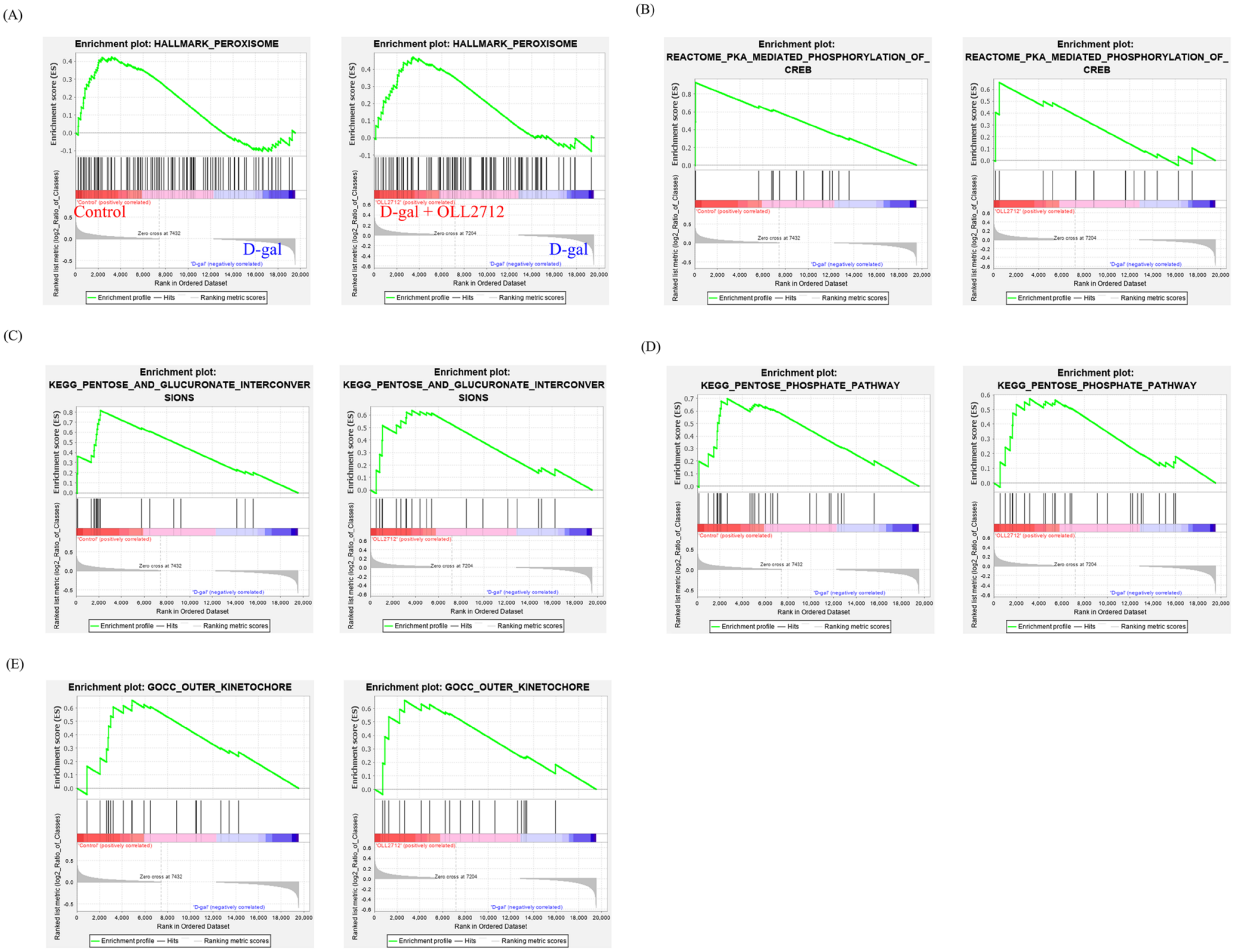
OLL2712 activated gene sets related to the anti‐oxidative response, metabolism, and cell division. Caco‐2 monolayers were treated with D‐gal alone or with D‐gal and OLL2712 simultaneously for 3 h. Gene set enrichment analysis was also performed. (A) Hallmark_Peroxisome, (B) PKA_Mediated_Phosphorylation_of_CREB, (C) Pentose_and_Glucronate_Interconversions, (D) Pentose_Phosphate_Pathway, and (E) Outer_Kinetochore. Comparisons between the control group and D‐gal (left panel) and D‐gal and D‐gal + OLL2712 (right panel) are shown.

By GSEA, the expression levels of metabolism‐ and antioxidant‐related gene sets were altered. D‐gal stimulation negatively enriched the gene set “peroxisome” in Hallmark (Normalized enrichment score; NES = 1.33, *p* = 0.06, *q* = 0.01), “PKA‐mediated phosphorylation of CREB” in the reactome (NES = 2.16, *p* = 0.00, *q* = 0.00), “pentose and glucuronate interconversion” (NES = 1.84, *p* = 0.00, *q* = 0.006), and “pentose phosphate pathway” (NES = 1.74, *p* = 0.00, *q* = 0.006) in KEGG compared with those in the control group. On the other hand, OLL2712 significantly suppressed or showed a tendency to suppress them, “peroxisome” in Hallmark (NES = 1.42, *p* = 0.009, *q* = 0.15), “PKA‐mediated phosphorylation of CREB” in the reactome (NES = 1.45, *p* = 0.05, *q* = 0.85), “pentose and glucuronate interconversion” (NES = 1.41, *p* = 0.07, *q* = 0.61), and “pentose phosphate pathway” (NES = 1.35, *p* = 0.07, *q* = 0.48) in KEGG compared to those in the D‐gal group (Figure 4A–D). About “pentose and glucuronate interconversion” and “pentose phosphate pathway,” we measured the expression levels of several selected genes involved in the pathways using qPCR. Contrary to our expectations, although the expression of some genes in the D‐gal group was altered, mostly decreased compared with the Control group, there was no significant recovery in the D‐gal + OLL2712 group (Table S1). Furthermore, D‐gal stimulation negatively enriched “outer kinetochore” in the GOCC (NES = 1.48, *p* = 0.03, *q* = 0.64) and OLL2712 suppressed it (NES = 1.44, *p* = 0.03, *q* = 0.83). “Outer kinetochore” is a region that mediates interactions between kinetochores and microtubules, which are important for cell division. In addition to immunofluorescence staining for senescence markers, microarray analysis revealed that D‐gal promotes cellular senescence and that OLL2712 suppresses senescent pathways (Figure 4E).

## 4 Discussion

It has been reported that some reagents, other than D‐gal, induce cellular senescence in vitro, such as doxorubicin (Wen et al. 2021) or hydroxyurea (Yu and Cheng 2021). These reagents inhibit cellular DNA synthesis and induce senescence (Petrova et al. 2016). In Caco‐2 monolayers, because the cells were expected to be in a steady state without active proliferation, we expected that these reagents would not work effectively. Therefore, D‐gal, which has a different mechanism of action, was selected for this study. D‐gal downregulated the protein level of pRB and upregulated the level of γ‐H2A.X, a marker of cellular senescence. At the gene expression level, *p53* and *p21* levels increased (Figure 1). Therefore, our results suggest that D‐gal induces cellular senescence in Caco‐2 monolayers. On the other hand, the protein level of pRB and γ‐H2A.X suggested that OLL2712 suppressed cellular senescence induced by D‐gal (Figure 1). Contrary to our expectations, the expression levels of *p53* and *p21* were not affected by OLL2712. One possible reason is that pRB expression is suppressed during cellular senescence through a pathway that does not involve *p53* or *p21* (Kumari and Jat 2021). It should be considered that OLL2712 protects against cellular senescence, but not via *p53* and *p21*. H2A.X is important for the early response to DNA damage and is located upstream of the p53/p21 pathway (Fragkos et al. 2009). Therefore, inhibition of γ‐H2A.X is generally thought to result in the suppression of p53/p21. However, ataxia telangiectasia mutated (ATM) kinase, which responds to DNA damage, can phosphorylate p53 independently of H2A.X (Kang et al. 2005). It is suggested that cellular senescence progresses without the involvement of γ‐H2A.X and that p53/p21 are activated independently of γ‐H2A.X. Furthermore, lipoteichoic acid, which is present in the cell walls of *L. plantarum* (Fischer et al. 1990), activates Toll‐like receptor 2 (TLR2) (Wang et al. 2020). TLR2 possibly affects the p53/p21 pathway via extracellular signal‐regulated kinase (ERK) (Kumari and Jat 2021; Lv et al. 2024). Therefore, we suspected that the γ‐H2A.X inhibitory effect of OLL2712 had no effect on p53 and p21.

OLL2712 may have an antioxidant effect from the result of antioxidant marker gene expression and microarray analysis (Figures 3 and 4A,D). As mentioned previously, D‐gal induces cellular senescence through the induction of oxidative stress by hydrogen peroxide. In this study, the levels of the antioxidant markers, *NQO1*, *GPX2*, and *SOD2*, were not significantly decreased by D‐gal treatment. However, the D‐gal + OLL2712 group exhibited the highest gene expression levels compared with other groups. Ru et al. (2022) showed that cellular senescence was induced in pig‐derived intestinal epithelial cells using D‐gal, but oxidative stress markers did not significantly change. They demonstrated that signaling pathways may differ in different tissues; however, the reasons for this are unclear. In addition, we tried to measure the hydrogen peroxide concentration in Caco‐2 monolayers stimulated by D‐gal. However, the hydrogen peroxide levels were very low, and there was no difference among the groups including the control (data not shown). Hydrogen peroxide is often used as an oxidative stress inducer in intestinal epithelial cells (Zhuang et al. 2019), whereas the levels detected in our experiment were < 1/100 of the concentration typically used for the purpose. It can be rapidly degraded by intracellular catalase. Therefore, it is considered that our experimental conditions using D‐gal induce oxidative stress but do not induce sustained production of hydrogen peroxide. On the other hand, the microarray results showed that gene sets “peroxisome” and “the pentose phosphate pathway” were downregulated by D‐gal induced cellular senescence and upregulated by OLL2712. Peroxisomes contribute to the promotion of fatty acid oxidation and the decomposition of hydrogen peroxide (Nordgren and Fransen 2014). The pentose phosphate pathway produces NADPH, which works to convert oxidized glutathione back to the reduced form after it decomposes hydrogen peroxide (Harris and Brugge 2015). It is possible that the increased antioxidant‐related gene expression by OLL2712 removes hydrogen peroxide induced by D‐gal, thereby exerting an antioxidant effect. Furthermore, a previous study showed that OLL2712 reduced chronic inflammation and improved metabolic disorders in a mouse model of type 2 diabetes (Toshimitsu et al. 2016). In the same study, OLL2712 suppressed diacron‐reactive oxygen metabolites (d‐ROMs), which is an index of serum oxidative stress. A previous report also suggested that OLL2712 has a strong antioxidant effect.

In addition, the decline in intestinal barrier function is probably associated with cellular senescence through antioxidant activity, because suppression of oxidative stress is also important for maintaining and improving intestinal barrier function (Li et al. 2022). Thus, we suspected that OLL2712 suppresses intestinal barrier dysfunction associated with cellular senescence through its antioxidant effects. OLL2712 restored intestinal barrier dysfunction related to cellular senescence (Figure 2). It has been reported that OLL2712 improves the intestinal barrier dysfunction in high‐fat diet‐fed mice through an anti‐inflammatory effect (Wang et al. 2023). We also recently reported that OLL2712 strengthened the intestinal barrier function through inducing autophagy even in the in Caco‐2 monolayers (Watanabe‐Yasuoka et al. 2023). Both anti‐inflammatory effects and autophagy are important factors for antiaging (Arias et al. 2024; Singh et al. 2024). In this study, it is possible that functions such as anti‐inflammation or autophagy of OLL2712 suppressed intestinal barrier dysfunction associated with cellular senescence. The effects of LABs on intestinal barrier function during cellular senescence and aging have been observed in other reports. For example, Yu et al. (2023) showed that another strain of *L. plantarum* maintained the integrity of intestinal barrier function and promoted longevity in aged mice. Yang et al. (2020) showed that senescence‐accelerated mouse prone 8, which received a probiotic cocktail containing LABs, had improved TJ‐related protein expression. In our study, it was shown that the *L. plantarum* JCM1149^T^ also protected from the intestinal barrier dysfunction caused by D‐gal. It remains unclear which components of *L. plantarum* contributed to the effect, or whether other strains may exhibit stronger efficacy. These questions warrant further investigation.

Microarray results showed that the expression levels of gene sets related to metabolism and cell division were downregulated by D‐gal‐induced cellular senescence and restored by OLL2712 (Figure 4B–E). PKA‐mediated phosphorylation of CREB is associated with the production of cyclic adenosine monophosphate, which serves as a substrate for energy production and is required for the differentiation of intestinal epithelial cells (Kabeya et al. 2018). In relation to pentose and glucuronate interconversions, glucuronate enters the pentose phosphate pathway after it is used for detoxification in the liver (Hankes et al. 1969). The pentose phosphate pathway is involved in nucleic acid synthesis and fatty acid biosynthesis (Stincone et al. 2015). It is possible that cellular senescence weakens the signals related to energy production, and OLL2712 restores them. Another report also showed that intracellular metabolism decreases due to cellular senescence (Roger et al. 2021), supporting our results. It is unfortunate that the expression levels of genes in the pathways related to metabolic regulation, which were found to be significantly altered in the microarray analysis, did not show significant differences under the effect of OLL2712 when measured by qPCR (Table S1). Although some gene expressions in the D‐gal group were decreased compared to the Control group, there was no recovery in the D‐gal + OLL2712 group. Although there was no statistically significant difference between the D‐gal group and the D‐gal + OLL2712 group, some genes showed higher expression levels in the D‐gal + OLL2712 group than in the D‐gal group. Therefore, the significance observed in the microarray analysis may have resulted from the combined expression patterns of multiple genes within the pathways, which might not have been detectable by examining individual genes alone. It is considered that the microarray analysis revealed significance as a pathway based on the level of multiple genes in the pathways. The outer kinetochore involved in cell division was also decreased by D‐gal treatment, but the Caco‐2 cells used in this study were monolayered, and we expected that cell division would not be so active. However, the result that genes related to cell division were active even under such monolayer conditions suggests that OLL2712 has a positive effect even on cells that are in a static state.

A limitation of this study is the use of Caco‐2 cells, which only allow the evaluation of absorptive epithelial cells. Cellular senescence in intestinal epithelial cells is thought to have a major impact on stem cells, which give rise to various cells, such as goblet cells and Paneth cells. It has been reported that intestinal organoids generated from aged mice have a worse shape than those generated from young mice, and the number of dividing organoids decreases with passages. The impairment of Wnt signaling with age has also been shown to reduce stem cell function and tissue regeneration (Nalapareddy et al. 2017). In addition to stem cells, gut microbiota also have a large impact. It is said that not only the intestinal barrier function but also the intestinal microbiota changes with aging. The changes in the microbiota lead to the inflammatory state and systemic symptoms. It has been reported that transplanting the microbiota of young mice into aged mice improves these conditions (Parker et al. 2022; Sovran et al. 2019). Previous studies showed that ingestion of OLL2712 changed the microbiota in elderly people and mice (Sakurai et al. 2022; Wang et al. 2023), and it is expected that it may have the effect of inhibiting cellular senescence through changes in the microbiota. However, in vitro approaches for evaluating coculture of intestinal epithelial cells and gut microbiota are still under development globally and lack standardized methodologies. In this study, we investigated the state of absorptive epithelial cells after cellular senescence was induced by D‐gal. In future studies, we will investigate the effects of OLL2712 on stem cells using induced pluripotent stem cell‐derived intestinal epithelial‐like cells and try coculturing intestinal epithelial cells with gut microbiota and OLL2712.

In conclusion, we showed that OLL2712 ameliorated the intestinal dysfunction associated with D‐gal‐induced cellular senescence in Caco‐2 monolayers. These results suggest that OLL2712 intake contributes to antiaging and the promotion of overall health by maintaining the function of the intestinal epithelial cells. We discovered the possibility that paraprobiotics contribute to the antiaging effects. For example, OLL2712 is used as an antiaging reagent. Even when mixed into a yogurt, OLL2712 retains its beneficial effects, such as improving glucose and lipid metabolism disorders (Toshimitsu et al. 2021, 2024). Although further clinical trials are required to clarify the role in cellular senescence, OLL2712 is expected to be a functional food ingredient suitable for daily consumption.

## Author Contributions

**Yumiko Watanabe‐Yasuoka:** conceptualization (equal), data curation with Caco‐2 cells (equal), formal analysis (equal), investigation (equal), methodology (equal), software (equal), validation (equal), visualization (equal), writing – original draft (equal). **Reiko Watanabe:** data curation with Caco‐2 cells (equal), methodology (equal), resources (equal), validation (equal). **Ayako Gotou:** data curation (equal), methodology (equal), resources (equal), validation (equal). **Haruyo Nakajima‐Adachi:** conceptualization (equal). **Satoshi Hachimura:** conceptualization (equal). **Toshihiro Sashihara:** conceptualization (equal), project administration (equal), supervision (equal), writing – review and editing (equal).

## Ethics Statement

The authors have nothing to report.

## Conflicts of Interest

Y.W.‐Y., R.W., and T.S. are employees of Meiji Holdings Co. Ltd. A.G. is an employee of Meiji Co. Ltd.

## Supporting information


**Figure S1:** fsn371019‐sup‐0001‐supinfo.docx. *Lactiplantibacillus plantarum* JCM 1149^T^ suppressed D‐gal induced reduction of TEER in Caco‐2 monolayers as well as OLL2712.
**Table S1:** Effects of OLL2712 on the relative expression of metabolic regulation‐related genes in D‐galactose‐induced senescent Caco‐2 cells.

## Data Availability

Data will be made available on request.
