# Supplementary material for: Lactiplantibacillus plantarum OLL2712 Protects From the Intestinal Dysfunction in D‐Galactose Induced Senescent Cells
Source: Food Sci Nutr. 2025 Sep 28;13(10):e71019. doi: 10.1002/fsn3.71019 (PMC12477272; doi:10.1002/fsn3.71019)
Supplement: Supplementary file 1 — Figure S1: fsn371019‐sup‐0001‐supinfo.docx. Lactiplantibacillus plantarum JCM 1149T suppressed D‐gal induced reduction of TEER in Caco‐2 monolayers as well as OLL2712. Table S1: Effects of OLL2712 on the relative expression of metabolic regulation‐related genes in D‐galactose‐induced senescent Caco‐2 cells. [file FSN3-13-e71019-s001.docx]

**Appendices**

Supplementary Figure 1. *Lactiplantibacillus plantarum* JCM 1149^T^ suppressed D-gal induced reduction of TEER in Caco-2 monolayers as well as OLL2712.

Caco-2 monolayers were treated with D-gal alone or with D-gal and either OLL2712 or *L. planatarum* JCM 1149^T^. Transepithelial electrical resistance (TEER) was measured. Data are presented as mean value ± standard error (n = 4, 5). Comparisons were performed by Student’s t-test with Bonferroni’s correction. ***; p < 0.001, *; p < 0.05.

**Appendices**

Supplementary Table 1. Effects of OLL2712 on the relative expression of metabolic regulation-related genes in D-galactose-induced senescent Caco-2 cells.

| *Genes* | Control | | |  | D-gal | | |  | D-gal + OLL2712 | | |
| --- | --- | --- | --- | --- | --- | --- | --- | --- | --- | --- | --- |
| *AKR1B1* | 1.00 | ± | 0.06 |  | 1.24 | ± | 0.05 |  | 1.25 | ± | 0.04 |
| *UGT2B7* | 1.00 | ± | 0.03 |  | 0.77 | ± | 0.10 |  | 0.81 | ± | 0.09 |
| *UGT2B10* | 1.00 | ± | 0.08*** |  | 0.68 | ± | 0.08 |  | 0.54 | ± | 0.03 |
| *UGT2B4* | 1.00 | ± | 0.13 |  | 0.96 | ± | 0.08 |  | 0.98 | ± | 0.06 |
| *UGT2A3* | 1.00 | ± | 0.12 |  | 0.99 | ± | 0.16 |  | 0.89 | ± | 0.14 |
| *G6PD* | 1.00 | ± | 0.04** |  | 1.37 | ± | 0.14 |  | 1.42 | ± | 0.14 |
| *RBKS* | 1.00 | ± | 0.05** |  | 0.77 | ± | 0.07 |  | 0.81 | ± | 0.07 |
| *ALDOB* | 1.00 | ± | 0.05** |  | 0.85 | ± | 0.04 |  | 0.82 | ± | 0.04 |
| *PGM1* | 1.00 | ± | 0.05 |  | 0.97 | ± | 0.09 |  | 0.99 | ± | 0.10 |

Data are presented as mean value ± standard error (n = 7). Comparisons were performed using Dunnett’s multigroup comparison test with D-gal as the reference. ***: p < 0.01, **: p < 0.05. Genes abbreviations: *AKR1B1*: aldo-keto reductase family 1 member B; *UGT2B7*: UDP glucuronosyltransferase family 2 member B7; UGT2B10: UDP glucuronosyltransferase family 2 member B10; *UGT2B4*: UDP glucronosyltransferase family 2 member B4; *UGT2A3*: glucronosyltransferase family 2 member A3; *G6PD*: glucose-6-phosphate dehydrogenase; *RBKS*: ribokinase; *ALDOB*: aldolase, fructose-bisphosphate B; *PGM1*: phosphoglucomutase 1. The same experimental methods as described in Section 2.3, "RNA isolation and quantitative polymerase chain reaction (qPCR)," were applied. The nucleotide sequence of the primers used in the quantifications were as follows:

AKR1B1: (F) TTTTCCCATTGGATGAGTCGG; (R) CCTGGAGATGGTTGAAGTTGG,

UGT2B7: (F) GATCCCAACAACTCATCCGCT; (R) CAGCAGCTCACTACAGGGAA,

UGT2B10: (F) GAAATGGACTACAGTTCTGCTGA; (R) GTGGATGAGTCGTTGGGATCA,

UGT2B4: (F) CAAATGTTGAGTTCGTTGGAGGA; (R) CTGACGTGTTACTGACCATCG,

UGT2A3: (F) GCCTTCGTTAATTGACTACAGGA; (R) GTTGATAAGCCTGGCAAGACAT,

G6PD: (F) CGAGGCCGTCACCAAGAAC; (R) GTAGTGGTCGATGCGGTAGA,

RBKS: (F) ATGGTCTGCCAGCTCGAAATA; (R) GAGAGGGTGTAGAACTGGGGA,

ALDOB: (F) GGCAGTTCCGAGAAATCCTCT; (R) CTCCTTGGTCTAACTTGATTCCC,

PGM1: (F) GATGGGGATCGAAACATGATTCT; (R) GCTGGAAATACGGAATGCTGAA.

F and R represent forward and reverse, respectively.
